# Can incubators work in Africa? Acorn Technologies and the entrepreneur-centric model

**DOI:** 10.1186/1472-698X-10-S1-S7

**Published:** 2010-12-13

**Authors:** Justin Chakma, Hassan Masum, Peter A Singer

**Affiliations:** 1 McLaughlin-Rotman Centre for Global Health, University Health Network and University of Toronto, 101 College Street Suite 406, Toronto ON, M5G 1L7, Canada

## Abstract

**Background:**

Incubators are organizations that support the growth of new and typically technology-based enterprises, by providing business support services that bring together human and financial capital. Although the traditional role of incubators has been for economic development, they may also be a useful policy lever to tackle global health, by fostering the development and delivery of local health innovation.

Given its high disease burden, life sciences incubators hold particular potential for Africa. As the most industrially advanced African nation, South Africa serves as a litmus test for identifying effective incubator policies. The case study method was used to illustrate how one such publicly funded incubator founded in 2002, Acorn Technologies, helped to catalyze local health product innovation.

**Discussion:**

Acorn helped to support twelve biomedical device firms. One of them, Real World Diagnostics, was founded by a trainee from Acorn’s innovative internship program (Hellfire). It developed rapid strip diagnostic tests for locally prevalent diseases including schistosomiasis and HIV, and reported $2 million (USD) in revenue in 2009.

Acorn achieved this success by operating as a non-profit virtual incubator with little physical infrastructure. Employing a virtual model in combination with stringent selection criteria of capital efficiency for clients proved to be effective in reducing its own fixed costs. Acorn focused on entrepreneurship training and networking, both critical at an early stage in an environment dominated by multinational biomedical device companies.

Acorn and its clients learned that employing a cross-subsidy business model allowed one to generate royalty revenue through imports to subsidize R&D for local diseases. However, funding constraints and government expectations for rapid self-sustainability forced Acorn to merge with its sister biotechnology incubator in 2009.

**Summary:**

A key to Acorn’s achievements was identifying entrepreneurs with technologies with health and economic impact, and providing them with flexible support from an early stage. A virtual organizational model helped Acorn to focus on supporting entrepreneurs. Governments and funders may wish to consider incubation strategies that draw from these good practices. With the right policies and business models, incubators have the potential to generate both health and economic benefits for Africa.

## Background

The traditional role of incubators has been economic development [1], but there have been recent suggestions that they may be relevant to global health as well [2]. A growing literature illustrates the potential health benefits that can accrue from the development and delivery of locally-fostered innovation, and barriers to achieving these benefits [3].

As organizations dedicated to fostering early-stage innovation, incubators might help facilitate the establishment and growth of new health technology enterprises by providing business support services that bring together a critical mass of human and financial talent. Innovative African nations may be good candidates for employing incubators to tackle global health. Moreover, the potential business opportunity for private sector healthcare in Africa itself is significant – estimated at $35 billion by 2016 [4].

As the most industrially advanced country in Africa, South Africa serves as a litmus test for identifying good practices for incubating health technology in low-resource settings [5,6]. While levels of economic development are different, South Africa shares with other African nations similar challenges in balancing economic development with creating solutions for local health needs, as well as industry structures dominated by multinationals and export businesses with generally little private-sector R&D investment. There exists a stark contrast between the richness of South Africa’s biomedical research and the rarity of start-up companies commercializing and translating those health technologies. (See the introductory paper in this BMC series for discussion of this contrast in other African nations.)

In 2001, the South African government established several publicly funded incubators including Acorn Technologies to spur the development of life science ventures to tackle local health problems such as the HIV/AIDS epidemic [5,6]. Based in Cape Town, Acorn helped to create several successful biomedical device companies by operating as a non-profit virtual incubator with a core management staff of five that focused on entrepreneurship training without physical infrastructure. Yet, funding constraints with an annual budget of only $500 000 USD and government expectations for rapid self-sustainability led to reduced service offerings, and an eventual merger with its sister incubator Cape Biotech Trust in 2009.

We used a case study design. Our analysis is based on semi-structured interviews with key informants, site visits in South Africa, and literature analysis. We conducted interviews with informed consent of personnel from Acorn Technologies, Cape Biotech Trust, DISA Vascular and Real World Diagnostics between October 2007 and June 2009, along with follow-up discussions. We also analyzed background documents from peer-reviewed literature, news reports, books, government and NGO reports, and web sites. Representatives of Acorn Technologies and some of its investees were asked to fact-check the case study; the analysis and interpretation is our own. All quotes are from the interviews unless otherwise noted, and with permission. This study was approved by the Office of Research Ethics of the University of Toronto.

In this article, we explore the potential of incubators in the African context through a case study of one incubator based in South Africa: Acorn Technologies. We begin by describing Acorn’s formation, business model and successes, and merger with a sister incubator, noting key challenges and decision points in the process. We then analyze the Acorn case as a whole to suggest lessons learned for supporting early-stage innovation in low resource settings, especially for global health.

## Discussion

### Seeding South Africa’s Life Sciences Sector

Based in Cape Town, Acorn was started in 2002 by a consortium composed of the University of Cape Town, University of Stellenbosch, Catalyst Innovations (a venture capital group) and the biomedical device pioneer DISA Vascular, under the auspices of a joint program between the Department of Trade and Industry (DTI), Department of Science and Technology (DST), and the European Union (EU). It hoped to ease the challenges of commercialization by providing business advisory services and networks of industry contacts to life sciences entrepreneurs, with a focus on biomedical devices. (See Table 1 for a timeline of Acorn Technologies.)

**Table 1:** History of Acorn Technologies

Year	Event
**2001**	South African government asks a consortium comprising a venture capital firm, a university tech transfer office, a university’s biomedical engineering department and a successful biomedical device company to start Acorn.
**2002**	Acorn Technologies is incorporated as a section 21 not-for-profit organization and receives initial grant funding for three years from the Department of Trade and Industry, Department of Science and Technology and the European Union.South African government releases a set of recommendations in the report “A National Biotechnology Strategy for South Africa” including the creation of biotech incubators
**2003**	Department of Science and Technology withdraws from Acorn Technologies in order to focus on creating biotechnology regional innovation incubators, including Cape Biotech Trust
**2005**	Acorn launches its first and only run of its successful Hellfire internship program to identify potential scientists and train them to be entrepreneurs. The program closes afterwards due to lack of funding.
**2006**	Acorn, Cape Biotech Trust and the Medical Research Council sign a memorandum agreeing to work more closely together and form the “Kopano” partnership to facilitate technology commercialization
**2007**	Acorn approaches Cape Biotech Trust to discuss merging and secures funding for medical device projects from the Department of Science and Technology. Merger is completed by the end of the year.
**2008**	Medical Device Center of Competence is created by the South African government within Cape Biotech Trust to maintain biomedical device support services

According to Acorn’s CEO Craig Landsberg, the biomedical device sector in South Africa is dominated by multinationals. These firms have pre-established relationships and distribution channels, which makes it challenging to find clinicians willing to be involved in the testing and local development of products. Success depends on building strong relationships with physicians through a sales force and the right distributor – aspects entrepreneurial scientists are often unfamiliar with.

Acorn aimed to serve as an intermediary between granting agencies like the Medical Research Council and late-stage investors such as Cape Biotech Trust (a South African investor of public funds for life sciences ventures) to help early stage start-ups create business strategies, register patents and procure funding from other government and external sources [6]. Rather than try to provide every service in-house, Acorn operated as a virtual incubator that outsourced business services such as market analysis and auditing. This allowed Acorn to focus on its core competency of mentoring entrepreneurs and connecting them to the right people or organizations. Acorn’s virtual model was partly due to limited funding, but was also a conscious decision to focus on mentoring early-stage entrepreneurs.

As a non-profit organization, Acorn did not aim to be self-sustaining financially, but rather to provide the resources necessary for biomedical device entrepreneurs to succeed. The overarching goal was to build sustainable businesses with innovative technologies with both financial and local health impact, in line with the mandate of the DST. In exchange for services, Acorn typically took either a percentage of royalties or an equity stake, which ensured self-selection of serious entrepreneurs. Any returns on investments were re-invested into new start-ups.

### Finding leads and potential clients

Although Acorn received almost 300 business proposals from potential scientist-entrepreneurs, it chose only a small number of high-quality companies to work with, and dedicated time and effort to ensuring their success.

The majority of Acorn’s leads for projects came from word-of-mouth and referrals. Acorn lacked the resources to actively pursue leads, but did have partnerships in place with local university technology transfer offices. This led to several projects for developing medical devices for surgery and infectious disease diagnostics. However, Acorn also advised service-oriented businesses for metallurgy, genetic testing, and electronic health records. See Table 2 for a list of Acorn’s investees.

**Table 2:** Acorn’s Portfolio of Incubatees

Company Name	Type of Company	Target Market/Product
Gknowmix	Genomics	Genetic Testing and Counselling
Biovac Institute (from CBT)	Non-Profit Vaccines	Vaccines
Pointcare Technologies	Biomedical Devices	Diagnostics “Lab in Briefcase” for AIDS Therapy and Cardiac Arrest
One Eighty	Biomedical Device Consulting	Metallurgic Consulting Services for Biomedical Devices
SunBio (from CBT)	Molecular Biology	3^rd^ Generation Yeast Strains for Winemaking
Sinapi Biomedical	Biomedical Devices	Medical Devices for Thoracic Surgery
Real World Diagnostics	Biomedical Devices	Rapid Diagnostic Strip Tests
Elective Lifestyle	Insurance	Cosmetic Surgery
Femipap	Biomedical Devices	Medical Devices for Female Healthcare/Cervix Self-Sampling
Pin Sealer	Biomedical Devices	Negative Pressure Wound Therapy Device
Smart Surgicals	Biomedical Devices	Robotic System for Minimal Invasive Surgery
Surgical Consent	Medical Informatics	Online Consent Form Management

One such investee, Gknowmix, provided web-based genetic testing and counselling services to help physicians and patients manage cardiovascular disease by integrating clinical, pathological and lifestyle risk factors for a comprehensive assessment [6,7]. As of early 2010, Gknowmix offered two genetic tests for multifactorial conditions – the Cardiovascular Genescreen and the Wellness Genescreen – priced at roughly 4000 rand each [8].

Sourcing of projects from domestic industry was limited since manufacturing and distribution of imported products accounted for most of the local market. Acorn’s sister incubator Cape Biotech Trust, also located in Cape Town, outsourced some projects to Acorn. Engaging researchers directly at local universities is one strategy Acorn employed to identify more project leads. In one instance, Acorn collaborated with the University of Stellenbosch to develop biomedical device projects, including a non-invasive haemoglobin meter, into working prototypes.

A prominent example of Acorn’s virtual approach to engaging and identifying researchers was its Hellfire internship program. Business-oriented internship programs are rare for scientists. The program identified and placed promising young scientists into established local life science firms for a year, with supporting business skills courses at the University of Stellenbosch. Hellfire spawned the incubatee Real World Diagnostics, to which we now turn.

### Supporting the development of Real World Diagnostics

Acorn’s emphasis on soft skills and social impact helped develop its incubatee Real World Diagnostics. One intern in the Hellfire program, Ashley Uys, improved an existing test for HIV while simultaneously developing a new test for schistosomiasis. The host firm, Vision Biotech, allowed Mr Uys to spin off his own company under the condition that it would be the marketing agent for Real World’s products in public sector markets in which Vision did not compete. Vision helped Mr Uys register the company and develop a business strategy in 2006. At the age of 23, Mr Uys became a CEO.

Real World Diagnostics’ rapid strip tests for HIV, malaria and other locally endemic diseases were reported to be cheaper compared to laboratory techniques or machinery, with prices ranging from 3 to 20 rand ($0.36 - $2 USD) as of early 2009.

In starting Real World Diagnostics, the first problem was cash flow. Generating initial sales in the diagnostics market was challenging due to competing multinational companies with established networks of distributors.

At this point, Acorn advised Real World Diagnostics to import diagnostic products into the local market to generate revenue for R&D. Real World supplied pharmacies directly at a national level, and used distributors to supply hospitals, retail stores and wholesalers. Using revenue generated from importing products, as well as lead funding from the government, Real World was able to develop several new tests for niche markets. Mr Uys was able to offer these new tests at a cheaper price because they were manufactured locally at Vision Biotech’s cGMP facility.

As HIV, malaria, and pregnancy tests represent mass markets based on high volumes and low margins, new entrants require the ability to produce inexpensively, along with access to distribution channels which multinational firms often already control. To reduce risk and sustain local health impact, Real World moved beyond HIV, malaria and other mass market diagnostics to develop niche, novel diagnostics. Real World reported that it was collaborating with the University of Cape Town to develop a test for beak and feather disease, a contagious and fatal viral disease in birds; South Africa is one of the biggest parrot export markets in the world. Another product was a test for EtG (ethyl glucuronide), a marker for alcohol consumption. Developed through a joint venture between UK-based Trimega Laboratories and Real World Diagnostics, the EtG test allows detection of alcohol consumption for up to 24 hours after actual consumption [9,10].

Recently, Real World was alleged to have used contacts and science from the joint venture without consent, and was ruled against by the Western Cape High Court [11]. Despite these challenges, Real World Diagnostics had two full-time and ten part-time employees and reportedly generated revenues of $2 million USD in 2009.

### Dealing with political investment horizons

While successes such as Real World established Acorn as a legitimate resource for early-stage life science entrepreneurs, the politics of the national ministries threatened its existence. Acorn received its initial funding from a joint program between the Department of Trade and Industry (DTI), Department of Science and Technology (DST) and the European Union (EU). The program spanned a wide variety of industries including furniture manufacturing incubators. However, in 2003, the DST withdrew from the program to create its own set of biotech regional innovation centers including Cape Biotech Trust – thus creating redundancy and leaving the DTI, whose key imperative was job creation, as Acorn’s primary funder. Under the DTI, Acorn was just one of several incubators, many of which supported industries where innovations require far shorter time scales, such as information technology and floriculture.

Acorn’s funder, the DTI, provided only operational funding and not funds to invest into its clients. Acorn received only ~5 million rand (~$500 000 USD) in annual funding [12]. By contrast, the biotech regional innovation centers established by the DST in 2003 – including Acorn’s closest ‘rival’ Cape Biotech Trust, also based in Cape Town – each received approximately 40 million rand (~$4 million USD) per year to invest into biotech start-ups. Acorn’s CEO Craig Landsberg recalls that about 3 years into Acorn’s existence, the DTI started to expect self-sustainability from each of its incubators. With little understanding of the long timeframes in the life sciences, and little political incentive to change that understanding, the DTI reportedly pressured Acorn to generate income from its incubator activities. This caused a clash between revenue generation pressures and the reality that life sciences commercialization takes 5 to 12 years to break even. It led to the closure of the successful Hellfire internship program that had spawned Real World Diagnostics.

The DTI stated that they would fund Acorn only if it worked with companies already exporting internationally, which prompted Acorn to temporarily shift from supporting early-stage projects to supporting entities that were close to commercialization or already exporting internationally. The DTI approach might have allowed Acorn to become self-sustainable through royalties, but at the loss of its early-stage focus.

Acorn’s CEO Craig Landsberg developed a royalty-based model for Acorn’s early-stage clients, where it took a small percentage of revenues. The royalties allowed Acorn to appease the DTI by demonstrating some flow of revenue. Landsberg says the second, more important benefit of the royalty-based model was to avoid the sense of *“terrible self-entitlement”* that pervades South Africa with regards to government-funded services, which were not valued if free. All contracts with new clients had a royalty set at around 3.5-5% of revenues for a fixed period of no more than 18 months.

The model allowed flexibility around the timings of royalty payments, so as not to cash-strain clients, but nonetheless ensured accountability from entrepreneurs in meeting royalty payments. Flexible payments were especially important for firms developing products with social impact, since often these were products destined for public health markets where payment and thus revenue was delayed and unpredictable. Acorn’s virtual model also proved critical under the pressure of self-sustainability, allowing costs to be reduced by outsourcing services such as business plan writing and market analysis.

Ultimately, Acorn’s funding gap became too large. The DTI proposed a model where the DTI, the provincial government, and the incubator’s income would each cover one third of the operational expenses. The problem with this model was that provincial governments reportedly had little political interest in supporting projects unless they were technologies such as biofuels or plant biotechnology with clear economic and development impact for local communities; it was not clear how Acorn’s portfolio met these criteria. Another option, which Acorn contemplated seriously and eventually undertook, was to seek national funding from the DST and merge with Cape Biotech Trust.

### Merging with Cape Biotech Trust

At first glance, Cape Biotech and Acorn’s merger seemed natural given that they happened to have been established next door to each other in Cape Town. Cape Biotech had even outsourced incubation work to Acorn during its earlier days. However, a combination of competition, falling-out between leadership, and concerns about Acorn’s financial stability led to ceasing of such partnering. Moreover, Cape Biotech focused on late-stage companies, while Acorn continued with early-stage projects. But *“the rules of the game were changing,”* as the DTI demanded self-sustainability.

This financial uncertainty caused many researchers and entrepreneurs to stay away from Acorn’s services. Since Cape Biotech Trust had already started to put incubation services in place, Acorn’s board supported Acorn’s migration from the DTI into the DST by merging with Cape Biotech.

The value for Acorn was obvious. Although DTI was supportive of Acorn, DTI’s incubation model – focused on quota-based job creation, and sustainability over impact – didn’t fit the life sciences. Since Cape Biotech Trust did not yet formally have an incubation offering for its clients, the question was whether more value would be created for its clients through a merger. Some concerns involved Cape Biotech’s role as an investment vehicle as opposed to Acorn’s role as an advisory and support service, and the fact that Cape Biotech focused its attention strictly on biotechnology as opposed to Acorn’s focus on biomedical devices. This meant that Acorn’s clients were potentially at risk of losing support. But there was a consensus that the market might be too small for two organizations.

All of Acorn’s late stage projects were therefore transferred to Cape Biotech, while Acorn received a period of 12 months of funding at between 30-50% to ensure continuity of projects that Cape Biotech could not absorb into its portfolio.

The merger left the fate of the biomedical device clients uncertain. Fortunately, the DST was in the process of establishing a number of Centers of Competence. One of them happened to be a biomedical device center, which was placed inside the newly merged organization. Acorn’s staff was hired into this Center. The primary functions of the new organization were meant to include skills development, incubation, networking and clustering. Acorn’s expertise in nurturing early-stage projects complemented Cape Biotech Trust’s late-stage projects by creating a pipeline of potential late-stage life sciences opportunities.

Cape Biotech Trust and the other biotechnology regional innovation centers, in turn, merged into a national initiative: the Technology Innovation Agency, which is expected to scale up to an annual budget of 1 billion rand ($130M USD) by 2013 [13]. A key function will be to provide funding for private sector companies to work with science councils on high-risk, early-stage research with support from existing incubation staff. The TIA is also tightly linked to national policy with its own staff allocated to advocacy and strategic relationships, as well as funding through grants, soft and convertible loans, and public-private partnerships.

While Acorn and Cape Biotech Trust no longer exist in name, many of their personnel and functions are still active, and their formative experiences have allowed South Africa to better understand and integrate incubators into its national innovation strategy.

### Lessons learned

The story of Acorn Technologies offers several lessons for innovation strategies in low-resource settings. Below we suggest some insights that entrepreneurs, policy makers, and practitioners may take.

#### Accept that life sciences may require long time horizons for commercialization

Incubators need flexibility in supporting early-stage life sciences ventures, which may require long timeframes to demonstrate success. In the face of pressure from the government to generate revenues, Acorn was forced to adopt a royalty-based model to demonstrate short-term focus. While the model bought Acorn additional time to wait for renewed funding or new opportunities, it was ultimately forced to merge with Cape Biotech Trust. Additional funding could have prevented the closure of the successful Hellfire internship program.

#### Screen clients stringently, and require evidence of focused commitment

Although Acorn received hundreds of business proposals from potential scientist-entrepreneurs, it chose only a small number of high-quality companies to work with, and dedicated time and effort to ensuring their success. Part of its selection approach was to insist that entrepreneurs agreed to give Acorn a royalty in exchange for services, so that only serious entrepreneurs would submit plans.

Stringent screening policies can improve graduation results, by improving resource allocation and accountability. As a point of comparison, Israel although having very successful enterprises has a low company survival rate of 53% [14] – its incubators are similar to South Africa’s in being government funded and non-profit. Not every discovery warrants its own company.

#### Enable hands-on entrepreneurial training and support

The experience of Acorn suggests that the importance of “soft” services such as networking and mentorship should not be underestimated. In the developing world, business power is often concentrated in a few incumbent firms [15]. Penetrating their stranglehold on distribution channels and human capital requires networking by entrepreneurs. As most scientists have little business experience, incubators must take a “hands-on” approach in developing business strategies or pairing scientists with entrepreneurs, and training both in marketing and presentation skills.

Acorn was able to provide its incubatees access to experienced life science entrepreneurs and legal and financial experts, who could provide services such as strategic advice, a strong board, fiscal discipline, and access to networks of connections. Acorn’s internship and business training programs for scientists keen to pursue the entrepreneurial route helped create a pipeline of potential talent for its start-ups. Indeed, Acorn helped to identify and train the founder of Real World Diagnostics, and provided him with the opportunity to work at Vision Biotech and gain private sector exposure. Real World Diagnostics, in turn, benefited from Acorn’s networks in establishing its distribution channels, meeting with international partners, and securing government grants. Governments may therefore wish to consider supporting internship programs such as Hellfire which can train scientific entrepreneurs, or partnering with non-profits which can do the same.

#### Consider a virtual organization model

Acorn’s decision to employ a virtual model and forgo providing many of its business services in-house streamlined the organization’s costs and services, and allowed it to focus on core competencies of mentorship and networking.

One question to ask might be, under which conditions is a virtual model appropriate? South Africa already has strong research facilities at its universities, so this may have made specialized physical infrastructure unnecessary. This may or may not be true in other developing nations and emerging economies. In some cases, investments in physical infrastructure are allocated for purposes other than health product development. In India, for example, entrepreneurs have complained that physical biotechnology parks tend to host primarily non-commercial research [16].

If physical infrastructure is unnecessary, the possibility arises of global incubator networks based on models such as Endeavor, a non-profit based in New York City which is dedicated to promoting entrepreneurship in emerging economies through mentorship and networking [17]. Indeed, one of Acorn’s co-founders – DISA Vascular, a stent innovator – was able to connect with local South African hospital CEOs that would have normally ignored its request, through the Endeavor network.

#### Focus on local health impact

Where disease burden is high, governments and funders gain if they support investments that can improve local health while being financially successful.

One of the challenges that Acorn faced was convincing the DTI to think beyond traditional metrics and view social impact as a criterion for success. Acorn invested in some companies whose products would not necessarily have high profit margins, but would tackle contributors to the local high disease burden such as HIV/AIDS.

One strategy used was a cross-subsidy business model where selling imported products generated revenue to subsidize innovative R&D. Real World Diagnostics executed this model to develop improved tests for HIV and schistosomiasis, as well as for alcohol consumption and beak and feather disease.

#### Coordinate innovation efforts across different governmental departments

The lack of coordination between the DTI and the DST contributed significantly to Acorn’s challenges. This suggests that governments and other partners should consider coordinating innovation efforts to avoid redundancy and inefficiencies.

Integration of support from early to late-stage functions in a single organization can help increase the chances of success by providing critical support and access to capital throughout the mid to late stage development process. It can also provide increased financial stability for the overall incubation effort, with royalties generated from late-stage investments that are presumably lower-risk subsidizing early-stage functions such as internship programs and start-ups. Support and protected time for early-stage companies is especially important for life sciences companies whose aim is health impact.

## Summary

Given that South Africa is more industrially advanced yet faces similar health and commercialization challenges to other African nations, learning from the challenges that Acorn faced may be helpful for other African stakeholders aiming to catalyze innovation relevant to local health problems. Both health and economic benefits are possible from investing in local health product innovation. Incubators may be one such vehicle to invest in, especially if implemented effectively and in contexts where sufficient scientific and financial capacity can be concentrated.

Acorn learned to overcome the challenges of bringing governmental and public support to the life sciences industry, and supporting prospective entrepreneurs with little business experience. The long time frames before life science companies are profitable made finding a sustainable model difficult, especially given Acorn’s desire to have both health and financial impact.

Acorn developed an entrepreneur-centric business model to tackle these issues and achieve its purpose of helping start-up companies. To fiscally maintain this model, Acorn ultimately had to reach out to sister organizations and integrate its services. Employing a virtual and entrepreneur-centric model in combination with policies of capital efficiency and stringent screening proved to be effective in reducing fixed costs, while simultaneously allowing focus on networking and mentoring.

The key emphasis was on soft skills. Internship programs such as Hellfire illustrate how entrepreneurial scientists can be identified early and trained to grow companies. Networking was likewise critical in an environment dominated by multinational biomedical device companies. Such training programs could be implemented at relatively low cost in other African countries, but require engagement of existing firms and experts. Where the requisite experience does not exist locally, there may be opportunity for international networking and mentorship.

Governments might incentivize existing firms to adopt cross-subsidized import/R&D business models to tackle local diseases. Finally, African countries with similar scientific strengths may consider pooling resources together to create “regional innovation communities” with incubators as organizing bodies [18]. With the right policies and business models, incubators have the potential to generate economic and health benefits for Africa.

## Competing interests

None declared.

## Authors' contributions

JC, HM and PAS contributed to the concept and design of this study, analyzed the findings, and participated in manuscript development. HM participated in site visits.
